# Low costs and opportunities for efficiency: a cost analysis of the first year of programmatic PrEP delivery in Kenya’s public sector

**DOI:** 10.1186/s12913-021-06832-3

**Published:** 2021-08-16

**Authors:** Kathryn Peebles, Kenneth K. Mugwanya, Elizabeth Irungu, Josephine Odoyo, Elizabeth Wamoni, Jennifer F. Morton, Kenneth Ngure, Elizabeth A. Bukusi, Nelly R. Mugo, Sarah Masyuko, Irene Mukui, Jared M. Baeten, Ruanne V. Barnabas, for the Partners Scale-Up Project Team

**Affiliations:** 1 Department of Epidemiology, University of Washington, Seattle, WA USA; 2 International Clinical Research Center, HMC # 359927 325 Ave, WA 98104 Seattle, USA; 3 Division of Disease Control, School of Public Health, Makerere University, Kampala, Uganda; 4 Department of Global Health, University of Washington, Seattle, WA USA; 5 Partners in Health and Research Development, Nairobi, Kenya; 6 Centre for Microbiology Research, Kenya Medical Research Institute, Nairobi, Kenya; 7 Department of Public and Community Health, Jomo Kenyatta University of Agriculture and Technology, Nairobi, Kenya; 8 Department of Obstetrics and Gynecology, University of Washington, Seattle, WA USA; 9 National AIDS & Sexually Transmitted Infection Control Programme, Ministry of Health, Nairobi, Kenya; 10 Department of Medicine, University of Washington, Seattle, WA USA

**Keywords:** Pre-exposure prophylaxis, Health economic evaluation, Cost analysis, Public sector implementation

## Abstract

**Background:**

In 2017, the Kenyan Ministry of Health integrated provision of pre-exposure prophylaxis (PrEP) into public HIV-1 care clinics as a key component of the national HIV-1 prevention strategy. Estimates of the cost of PrEP provision are needed to inform the affordability and cost-effectiveness of PrEP in Kenya.

**Methods:**

We conducted activity-based micro-costing from the payer perspective to estimate both the financial and economic costs of all resources and activities required to provide PrEP in Kenya’s public sector. We estimated total and unit costs in 2019 United States dollars from a combination of project expense reports, Ministry of Health training reports, clinic staff interviews, time-and-motion observations, and routinely collected data from PrEP recipient files from 25 high-volume HIV-1 care clinics.

**Results:**

In the first year of programmatic PrEP delivery in 25 HIV-1 care clinics, 2,567 persons initiated PrEP and accrued 8,847 total months of PrEP coverage, accounting for 2 % of total outpatient clinic visits. The total financial cost to the Ministry of Health was $91,175, translating to an average of $10.31 per person per month. The majority (69 %) of financial costs were attributable to PrEP medication, followed by administrative supplies (17 %) and training (9 %). Economic costs were higher ($188,584 total; $21.32 per person per month) due to the inclusion of the opportunity cost of staff time re-allocated to provide PrEP and a proportional fraction of facility overhead. The vast majority (88 %) of the annual $80,811 economic cost of personnel time was incurred during activities to recruit new clients (e.g., discussion of PrEP within HIV-1 testing and counselling services), while the remaining 12 % was for activities related to both initiation and maintenance of PrEP provision (e.g., client consultations, technical advising, support groups).

**Conclusions:**

Integration of PrEP provision into existing public health HIV-1 care service delivery platforms resulted in minimal additional staff burden and low incremental costs. Efforts to improve the efficiency of PrEP provision should focus on reductions in the cost of PrEP medication and extra-clinic demand creation and community sensitization to reduce personnel time dedicated to recruitment-related activities.

**Trial registration:**

ClinicalTrials.gov registration NCT03052010. Retrospectively registered on February 14, 2017.

**Supplementary Information:**

The online version contains supplementary material available at 10.1186/s12913-021-06832-3.

## Background

Despite recent advances in treatment and prevention, more than 1,000,000 new HIV-1 infections occurred in 2018 in sub-Saharan Africa [1]. HIV-1 pre-exposure prophylaxis (PrEP) is a highly effective prevention method, reducing the risk of HIV-1 acquisition by more than 90 % [2]. In 2017, the Kenyan Ministry of Health (MOH) therefore integrated PrEP provision into the public-sector healthcare system as a key component of the national HIV-1 prevention strategy, with PrEP roll-out targeted both geographically and by priority population in an effort to dedicate resources to areas and populations with the highest prevention benefit [3].

Estimates of the cost-effectiveness and affordability of PrEP to prevent new HIV-1 infections are essential to ensuring high-yield and sustainable prevention programs. Previous cost-effectiveness analyses of PrEP have produced a wide range of estimates, from cost-saving [4] to $65,610 per infection averted [5]. Variation in estimates were dependent on multiple factors, including the degree of risk among the modeled population [5], the existence of concurrently delivered interventions [6], and, critically, the assumed or estimated cost of PrEP [4, 7]. However, empirical cost estimates of PrEP provision are limited and typically originate within the context of demonstration projects [5, 8, 9], which are not necessarily reflective of MOH program implementation. Detailed cost estimates of public-sector provision of PrEP are needed to serve as the foundation of contextually relevant cost-effectiveness and budget impact analyses.

The recent expansion of PrEP provision within Kenya’s public sector offers an opportunity to estimate the government and donor costs of PrEP provision to inform cost-effectiveness and affordability of PrEP in Kenya, which may be applicable to other African countries rolling out PrEP. We therefore estimated the incremental cost of public-sector HIV-1 care clinic-based provision of PrEP in Kenya.

## Methods

We conducted activity-based micro-costing following the Global Health Cost Consortium Reference Case guidelines [10] nested within the Partners Scale-Up Project, an implementation project operating in partnership with MOH to catalyze and evaluate scale up of PrEP delivery in 25 high-volume public HIV-1 care clinics in central and western Kenya [11]. All PrEP service delivery is provided per Kenyan national guidelines [12] within an existing service delivery platform by HIV-1 care clinic staff (including data clerks, receptionists, peer educators, social workers, nurses, clinical officers, HIV-1 testing and adherence counsellors, and pharmacy technologists), with technical advising for PrEP delivery provided by project staff. Existing service delivery platforms for HIV-1 care are managed by county governments in partnership with a PEPFAR-funded supporting partner. HIV-1-negative persons are eligible to initiate PrEP if they report one or more HIV-1 risk factors, such as an HIV-1-positive partner, inconsistent condom use, and having greater than one sex partner [12]. At PrEP initiation, clients undergo HIV-1 testing; additional laboratory tests, including hepatitis B surface antigen and serum creatinine clearance, are recommended, but not required [12], and are paid for by clients. National guidelines recommend a quarterly follow-up visit schedule [12], though MOH has provisionally required monthly PrEP refills.

We estimated both the financial and economic costs of all resources and activities required to provide PrEP in Kenya’s public sector. Financial costs capture only the incremental expenditures required to provide PrEP, and therefore most closely approximate the additional burden of PrEP provision to the MOH budget, while economic costs facilitate cost comparisons across programs by also incorporating the opportunity cost associated with re-allocation of clinic staff time to PrEP-related services and a proportional fraction of facility overhead. We further estimated both the project and MOH cost of PrEP provision. Because the majority of services are delivered within an existing MOH service delivery platform, project and MOH costs are nearly equivalent, with the exceptions of the cost of initial training and technical advising. Initial training costs include offsite conference facility costs, while ongoing MOH trainings are healthcare facility-based. Additionally, project-based technical advising is intensive, with bi-weekly facility visits, while we assume that MOH technical advising will occur monthly within existing HIV-1 care technical advising. We estimated all costs from the payer perspective, and additionally estimate direct non-medical costs borne by PrEP clients.

We classify costs as those required at start-up of PrEP implementation (including stakeholder coordination, training, and demand creation) and recurrent costs (including PrEP medication and other supplies, clinic staff salaries, capital goods, and facility overhead). Items included in each cost category are described in detail in Additional File 1. We estimated total and unit costs from project expense reports and project and MOH training reports across the 25 included HIV-1 care clinics, and additionally conducted clinic staff interviews and time-and-motion observations of PrEP provision in eight clinics in central and western Kenya in two time periods in February 2018 and March 2019. We obtained clinic staff salaries from published Kenyan civil service salary scales, including benefits [13], and assume the minimum wage of unskilled workers to approximate clients’ lost wages due to time spent to access PrEP [14]. PrEP medication costs include commodity cost as well as importation and central storage and distribution fees. We included the cost of capital goods newly purchased for PrEP provision, and proportionally allocated health facility overhead costs according to the fraction of total daily services comprised by PrEP-related activities. We captured direct non-medical costs of PrEP provision in structured interviews with a sample of PrEP clients as the cost of transportation for clinic visits and lost wages for the duration of transportation and the clinic visit.

We estimated the total number of annual PrEP appointments and PrEP medication dispensed from routinely collected clinical data in each of the 25 included HIV-1 care clinics. PrEP provision was established in clinics in a stepped schedule from January 2017 to July 2017, and we include a year of data from each clinic’s first date of PrEP provision. The total cost of PrEP service delivery is calculated as the sum of all annualized start-up costs and recurrent costs incurred in the first year of PrEP provision. The average cost per person-month of PrEP is then calculated as the total annual cost divided by the total number of months of PrEP coverage. Total estimated costs include all observed visits, and we separately estimated the cost attributable to monthly refill visits as the cost of each additional refill visit multiplied by the number of refill visits (estimated as the total number of PrEP visits recorded in routinely collected clinical data beyond those expected in a quarterly follow-up visit schedule). Expenditures reported in Kenyan shillings are converted to United States dollars (USD) [15], and all costs are reported in 2019 USD [16]. The annualized cost of capital goods is discounted at a rate of 3 % [17], and we assumed a useful life of five years for all capital items. Unit costs, calculations, and time-and-motion observations used in this analysis are included in Additional File 2. All analyses were conducted in Excel (version 16.5, Microsoft, Redmond, WA) and R (version 3.5.0) [18].

Institutional review boards at the Kenya Medical Research Institute and the University of Washington approved this study, and clients participating in structured interviews provided written informed consent.

## Results

In the first year of programmatic PrEP delivery in 25 high-volume HIV-1 care clinics, 2,567 persons initiated PrEP, accruing 8,847 total months of PrEP coverage from January 2017 to July 2018. The majority of those who initiated PrEP in this setting were in serodiscordant relationships (2,224, 86.6 %), while the remainder reported risk factors including a high-risk sex partner of unknown HIV-1 status (11.2 %), inconsistent or no condom use (6.7 %), and/or having greater than one sex partner (5.8 %).

### Financial and economic costs of PrEP provision

**Fig. 1:**
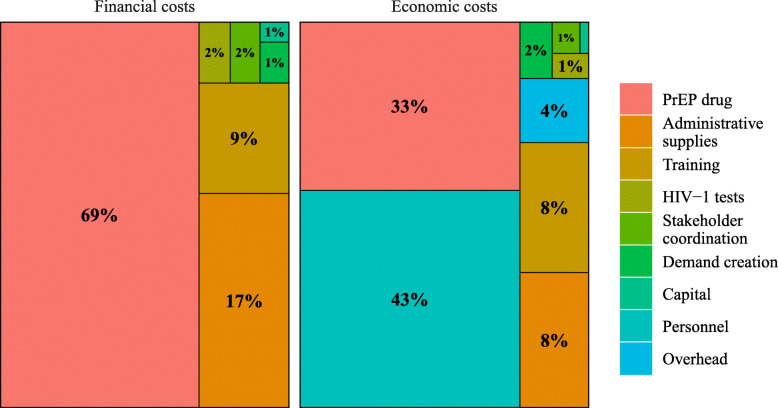
Distribution of financial and economic costs to the Ministry of Health in the first year of public sector PrEP provision in Kenya

The total annual financial cost to the MOH of PrEP provision across the 25 clinics was $91,175, with PrEP medication accounting for the majority (69 %) of the total cost, followed by administrative supplies (17 %) and training (9 %) (Table 1; Fig. 1). This translates to an average cost of $10.31 per person-month of PrEP coverage, with $7.09 of monthly costs attributable to PrEP medication. Within the Partners Scale-Up Project, financial costs were higher due to more intensive technical advising and offsite training expenditures (a total additional amount of $20,101 and $14,620, respectively), translating to an average cost of $14.23 per person-month of PrEP coverage.

**Table 1:** Total and unit Kenyan Ministry of Health costs of PrEP provision in Kenyan public HIV-1 care clinics (2019 USD)

	Financial costs			Economic costs		
	Total annual cost	Cost per PrEP client(*n* = 2,567)	Cost per person-month of PrEP(*n* = 8,847)	Total annual cost	Cost per PrEP client(*n* = 2,567)	Cost per person-month of PrEP(*n* = 8,847)
**Start-up**						
Stakeholder coordination	1,485	0.58	0.17	1,481	0.58	0.17
Training	8,147	3.17	0.92	15,162	5.91	1.71
Demand creation	981	0.38	0.11	3,096	1.21	0.35
**Recurrent**^a^						
Personnel	0	0.00	0.00	80,811	31.48	9.13
Supplies						
PrEP medication	62,700	24.43	7.09	62,700	24.43	7.09
HIV-1 tests	1,562	0.61	0.18	1,561	0.61	0.18
Administrative	15,813	6.16	1.79	15,813	6.16	1.79
Capital	487	0.19	0.06	487	0.19	0.06
Overhead	0	0.00	0.00	7,470	2.91	0.84
**Total**	**$91,175**	**$35.52**	**$10.31**	**$188,584**	**$73.46**	**$21.32**

^a^Recurrent costs include costs incurred across all visits types (initiation, follow-up, and PrEP refill)

The total annual economic cost to the MOH of $188,584 additionally includes the opportunity costs represented by staff time re-allocated to provide PrEP and a proportional fraction of facility overhead. PrEP medication and personnel accounted for similar proportions of economic costs (33 and 43 %, respectively), with remaining categories each accounting for less than 10 % of total costs (Fig. 1). Across 8,847 months of PrEP coverage, the average economic cost per person-month of PrEP was $21.32. Inclusion of higher project costs increases the total economic cost to $222, 530 and the average economic cost per person-month of PrEP to $25.15.

### Personnel time and costs of PrEP delivery

**Fig. 2:**
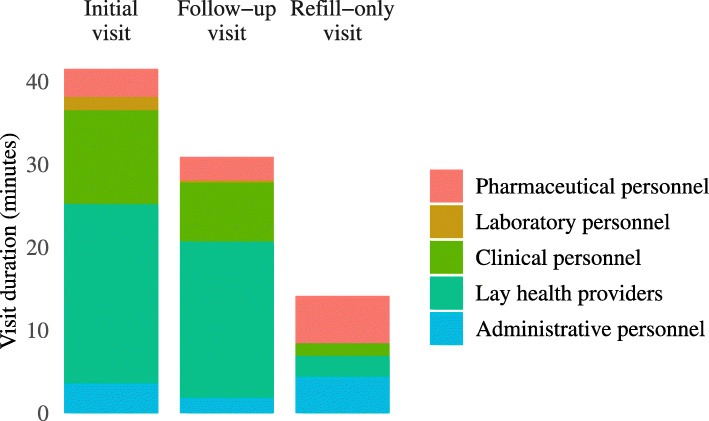
Staff time dedicated to PrEP provision at initial, follow-up, and refill-only visits

**Fig. 3:**
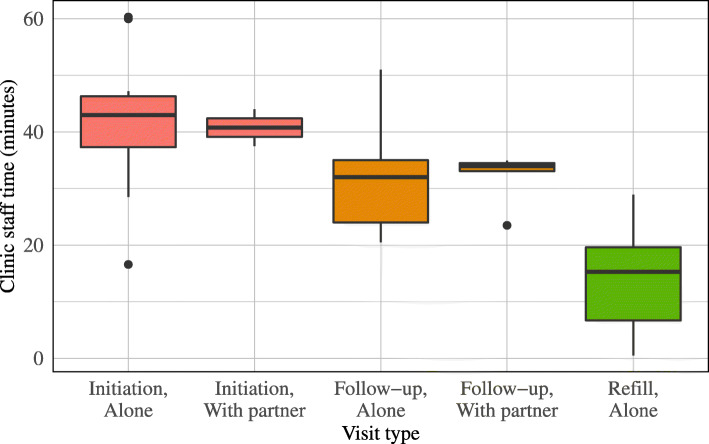
Distribution of total clinic staff time by initiation and follow-up visits attended by the PrEP client alone or with their HIV-positive partner

We completed 46 time-and-motion observations, including 14 PrEP initiation visits, 26 follow-up visits, and 6 refill-only visits. PrEP initiation visits required 43 min (IQR: 38, 46) of staff time, with clinical consultation accounting for approximately one-quarter of total time. Follow-up appointments had a median duration of 33 min (IQR: 24, 35), with the majority of services (19 min, 61 %) provided by lay health workers, such as peer educators and counsellors. Refill-only visits were considerably shorter, requiring 15 min of staff time each (IQR: 7, 20) (Fig. 2). PrEP clients accounted for approximately 2 % of total daily client volume in HIV-1 care clinics. The majority of clients (39, 85 %) attended clinic appointments alone, while the remaining 7 clients attended clinic with their HIV-positive partner to receive PrEP services concurrent to their partner’s receipt of antiretroviral therapy (ART) services. Clinic staff dedicated a similar amount of time to PrEP-related service provision activities when a partner was present and when the PrEP client attended the clinic alone (Fig. 3).

The vast majority (88 %) of the annual $80,811 economic cost of personnel time was incurred during activities to recruit new clients (e.g., discussion of PrEP within HIV-1 testing and counselling services [HTS]), while the remaining 12 % was for activities related to both initiation and maintenance of PrEP provision (e.g., client consultations, technical advising, support groups). The economic cost of personnel time for laboratory tests was particularly low ($330), given that fewer than 5 % of PrEP clients received creatinine clearance or hepatitis B tests.

### Client-borne costs

Sixty-seven clients reported their cost of transportation and total time dedicated to accessing PrEP (6 initiation visits, 48 follow-up visits with a clinical consultation, and 13 follow-up visits for a PrEP refill without clinical consultation). The time and cost of transportation to clinics were somewhat higher in central Kenya than in western Kenya due to the typically higher costs encountered in urban settings (Table 2). Clients spent approximately 3.0 h at the clinic for initiation (IQR: 2.3, 3.0) and follow-up (IQR: 1.0, 3.3) visits and 2.0 h (IQR: 1.0, 3.0) for refill-only visits. The total median cost per client, accounting for both the cost of transportation and the opportunity cost of the client’s time, was $7.11 (IQR: 4.26, 8.83) per initiation or follow-up visit in central Kenya and $6.11 (IQR: 2.87, 7.16) per initiation or follow-up visit in western Kenya. Similarly, the cost to clients per refill-only visit was somewhat higher in central ($5.97, IQR: 4.26, 8.54) than western Kenya ($4.97, IQR: 2.87, 6.87).

**Table 2:** Client time and costs of clinic attendance for PrEP services

	Time,median (IQR)(hours)	Cost,median (IQR)(2018 USD)
**Region**	**Transportation**	**Transportation**
Central	2.00 (1.12, 2.00)	$1.94 (1.02, 1.94)
Western	1.00 (0.75, 1.17)	$1.38 (0.97, 1.98)
**Visit type**	**Clinic**^a^	
Initiation	3.00 (2.25, 3.00)	--
Follow-up	2.88 (1.00, 3.27)	--
PrEP refill	2.00 (1.00, 3.00)	--

^a^Includes waiting and consultation time

### Additional costs attributable to monthly PrEP refills

Additional visits to provide monthly, rather than quarterly, refills represent an economic cost of $0.56 in personnel time per PrEP refill visit, for a total cost across 25 clinics of $531.51 in the first year of PrEP provision. Furthermore, each additional clinic visit costs an average of $5.40 (IQR: 3.06, 7.46) in transportation and lost wages to PrEP clients, translating to an average additional cost over six months of PrEP use of $16.20 (IQR: 9.18, 22.38) per client attending facilities that provide refills on a monthly basis.

## Discussion

In the first year of programmatic PrEP delivery in Kenyan public health HIV-1 care clinics, overall and per-client-per-month costs were low, at approximately $10 in incremental financial costs and $21 in incremental economic costs per month of PrEP coverage. This is the first costing analysis of public sector PrEP provision in HIV-1 care clinics, where the majority of PrEP clients in Kenya thus far have received services [19]. These cost estimates are therefore particularly relevant to inform additional research to determine if PrEP is cost-effective relative to the suite of available HIV-1 prevention interventions, the relative cost-effectiveness of PrEP provision across different service delivery points, estimation of affordability given Ministry of Health and donor budgets, and may also be useful to additional countries in sub-Saharan Africa considering PrEP scale-up.

Furthermore, this analysis highlighted important opportunities for increased efficiency in PrEP provision. PrEP medication was the primary cost driver, accounting for nearly three-quarters of the incremental financial cost of PrEP provision. Negotiation of lower prices may improve the financial feasibility of this highly effective prevention method. The Clinton Health Access Initiative reference cost for combination tenofovir disoproxil fumarate and emtricitabine is $6.05 (2019 USD) [20], a 7 % reduction compared to the cost of PrEP drug (exclusive of importation and distribution costs) incurred in this setting, suggesting room for reductions in drug costs. Efforts to reduce the financial cost of public sector PrEP provision may benefit from government-negotiated purchases of bulk administrative supplies, which were the second largest driver of costs when purchased as individual retail items within the Partners Scale-Up Project. The economic cost of personnel time was largely attributable to activities to recruit new clients, including discussion of PrEP within facility-wide HTS sessions. Additional community sensitization and mass media activities would likely reduce the amount of time needed to discuss PrEP in HTS sessions as PrEP access continues to scale up. These analyses also highlight low-cost program components that may play important roles in the success of PrEP delivery. For example, both demand creation and purchases of new capital items (including filing cabinets to aid in organization of new files and cellphone handsets to communicate with PrEP clients) accounted for only 1 % each of annual financial PrEP delivery costs, yet may increase uptake and facilitate the clinic’s ability to monitor and retain PrEP clients.

While PrEP is provided free of charge, clients incurred approximately $5–7 in direct non-medical costs per clinic visit, potentially representing an important barrier to PrEP uptake and continuation. The cost to clients of monthly refills was particularly high; efforts to enact a quarterly visit schedule would reduce the cost burden to clients, as well as, to a lesser extent, the healthcare system. Additionally, implementation efforts may benefit from streamlined PrEP delivery strategies, including expansion of PrEP services to additional platforms, such as lower-volume facilities and commercial pharmacies; differentiated care for PrEP; a semi-annual refill schedule with quarterly HIV-1 self-testing; and community-based PrEP provision.

Our estimates of the economic cost of PrEP delivery of $21.32 per person-month in this setting is similar to the estimated cost per person-month of PrEP provision in maternal and child health and family planning clinics in western Kenya ($26.52) [21]. Higher costs in the maternal and child health and family planning settings are predominantly due to the inclusion of creatinine testing for every client, whereas we observed very limited creatinine testing in HIV-1 care clinics. The cost of this test is typically paid by clients in this setting, representing a potential barrier to test completion. Future research should evaluate the cost-effectiveness of inclusion of creatinine clearance tests in the package of government-sponsored PrEP provision.

This analysis has several limitations. First, our estimates of recruitment-related costs, such as demand creation, do not include the cost of national PrEP campaigns, though these campaigns likely contributed to the demand for PrEP within HIV-1 care clinics. Second, the cost estimates presented here are based on annual PrEP enrollment of approximately 2,500 across 25 clinics, accounting for approximately 2 % of total client volume. In this setting, the additional work burden was readily absorbed by existing staff and facilities; as such, we did not estimate the economic cost of components such as building rental and supervision, as the proportional allocation of these existing resources to PrEP would be minimal. While we assume that reallocation of time to PrEP delivery does not impact ART delivery, the true opportunity cost of staff time is uncertain, and future analyses of the net cost of PrEP provision should account for any changes to ART delivery. Third, enrollment was relatively constant in the first three years of clinics included here (data not shown), though future uptake may be higher as existing barriers, such as low community awareness of PrEP and PrEP stigma [22, 23], are mitigated. While overall incremental financial expenditures would increase in such a scenario, the average cost per person per month of PrEP coverage is unlikely to vary much, given that the vast majority of monthly costs are attributable to variable, rather than fixed, cost items. Nevertheless, PrEP implementation remains a relatively new service, and program modifications in the future are likely. Cost estimates should be periodically updated to reflect such changes to maintain accurate budget impact and cost-effectiveness estimates.

## Conclusions

Integration of PrEP provision into existing public health HIV-1 care service delivery platforms resulted in minimal additional staff burden and low incremental costs. Efforts to improve the efficiency of PrEP provision should focus on reductions in the cost of PrEP medication and community sensitization activities to reduce personnel time dedicated to recruitment-related activities.

## Supplementary Information

**Additional file 1.**

**Additional file 2.**

## Data Availability

Cost data utilized in this analysis is included in Additional File 2. Additional datasets including individual-level participant data will be available after the parent study primary analysis is complete by contacting the International Clinical Research Center at the University of Washington at icrc@uw.edu.

## Abbreviations

ART: Antiretroviral therapy
HIV-1: Human Immunodeficiency Virus Type 1
MOH: Kenyan Ministry of Health
PrEP: Pre-Exposure Prophylaxis
USD: United States dollars
