# Supplementary material for: Low costs and opportunities for efficiency: a cost analysis of the first year of programmatic PrEP delivery in Kenya’s public sector
Source: BMC Health Serv Res. 2021 Aug 16;21:823. doi: 10.1186/s12913-021-06832-3 (PMC8365926; doi:10.1186/s12913-021-06832-3)
Supplement: Supplementary file 1 — Additional file 1. [file 12913_2021_6832_MOESM1_ESM.docx]

**Supplement Text S1**. Description of cost categories in project and Ministry of Health scenarios.

Start-up costs

*Training*

In the project scenario, training costs include the value of time of University of Washington-based personnel to develop training materials, the cost of conference facility fees and refreshments, the cost of training materials, including items such as picture frames to commemorate the training, the value of both trainer and trainee time, estimated according to the job group of each trainee, and the cost of transportation and lodging for both trainers and trainees. The Ministry of Health (MOH) scenario is equivalent with the exception that the cost of conference facility fees and trainee facilitation are excluded, as ongoing MOH trainings are facility-based. We additionally exclude the cost of miscellaneous items such as picture frames.

*Demand creation*

Demand creation costs include the value of time of University of Washington-based personnel to develop demand creation materials, the cost of printed posters and flyers, the value of donated airtime to broadcast PrEP-related information, the cost of meetings with church groups and trainings for members of the media, PrEP-branded lab coats for use in public health HIV care facilities, and cakes to reward clinics upon meeting PrEP enrollment milestones.

*Stakeholder coordination*

These costs include transportation reimbursement for attendees of meetings to coordinate PrEP-related activities in central and western Kenya. The value of attendee time is not included.

Recurrent costs

*Personnel*

Personnel time costs include personnel time for direct service delivery and other PrEP-related activities. The latter include PrEP discussions within HIV testing and counselling sessions, in facility-wide health talks, and in serodiscordant couple support groups. This category additionally includes the time for technical advising activities. In the project scenario, technical advising occurs weekly at each facility and project staff salaries are used. In the MOH scenario, we assume that technical advising takes place on a monthly basis within existing ART service delivery technical support and that technical advisors receive an MOH salary equivalent to that of a clinical officer.

*PrEP medication*

PrEP medication costs include a cost of $6.25 (2017 USD) per 30-day supply of PrEP donated by Gilead, as well as central storage and distribution fees and a one-time PrEP importation fee.

*HIV-1 tests*

The cost of HIV-1 tests is determined according to the cost per Determine testing kit (Abbott Diagnostics, Chicago, IL) and the quantity of HIV-1 tests included in each kit. Per Kenyan ART guidelines, HIV-1 tests are conducted at enrollment, the month one follow-up visit, and quarterly thereafter (1).

*Administrative supplies*

Administrative supplies include the cost of PrEP monitoring and evaluation tools, such as registries and diaries, airtime to contact PrEP clients, lab requisition books, refreshments for client support groups, and materials to open and maintain PrEP client files, including folders, client encounter forms, and appointment cards.

*Capital costs*

PrEP-specific capital costs are limited to the purchase of a new filing cabinet for each supported facility, cellphone handsets to call PrEP clients, and a blood pressure measuring device for a single facility that lacked one.

*Overhead costs*

Overhead costs include utilities (water and electricity) and maintenance and repairs.

Client costs

Client costs include the cost of round-trip transportation for clinic visits, as well as the opportunity cost of time spent on transportation and at the clinic. The opportunity cost of lost wages is valued at $1.14 per hour (2020 United States dollars), the minimum wage of unskilled workers (2). While clients bear the cost of guideline-recommended laboratory tests (creatinine clearance and hepatitis B virus) at most clinics, these tests were performed among fewer than 5% of clients, and so we do not include the cost of laboratory tests here.

**References**

1. Ministry of Health NASCPN. Guidelines on use of Antiretroviral Drugs for Treating and Preventing HIV Infection in Kenya 2016. Nairobi, Kenya: NASCOP; 2016.

2. Kandie P. The Regulation of Wages (Agricultural Industry). Kenya Gazette. 2017.
